# Collectanea Medica: Consisting of Anecdotes, Facts, Extracts, Illustrations, &c.

**Published:** 1821-02

**Authors:** 


### COLLECTANEA MEDICA:

CONSISTING OF

ANECDOTES, FACTS, EXTRACTS, ILLUSTRATIONS, &c.

*Relating to the History or the Art of Medicine, and the Auxiliary Sciences.*

Floriferis ut apes in saltibus omnia libant,

Omnia nos itidem depascimur aurea dicta.

*(This department is vacant, in consequence of the great and unusual extent of the Journal occupied by the* Original Communications *which it has been necessary to insert in the present Number.)*

